# Reasons for choice of glaucoma surgery in eyes not treated with anti-glaucoma medications

**DOI:** 10.1186/s12886-022-02369-z

**Published:** 2022-03-30

**Authors:** Kazunobu Sugihara, Hiroki Fukuda, Tetsuro Omura, Masaki Tanito

**Affiliations:** Department of Ophthalmology, Shimane University Faculty of Medicine, Izumo, Japan

**Keywords:** Glaucoma surgery, Primary angle closure disease (PACD), Goniosynechialysis (GSL), Cataract surgery, Minimally invasive glaucoma surgery (MIGS), Dementia, Poor adherence, Drug allergy

## Abstract

**Background:**

In the real world, some glaucoma patients can undergo an incisional glaucoma surgery without using medication. The rate of cases with no medication treatment at the time of surgery among those that underwent incisional glaucoma surgeries performed in our department was reported.

**Methods:**

The department database of Shimane University Hospital for eyes that underwent incisional surgeries to manage glaucoma at the hospital between April 2018 and September 2020 were searched. By reviewing the medical charts of 1,417 consecutive eyes listed, 90 (6.4%) eyes of 67 subjects (mean age of 72 ± 16 years; 22 men, 29 eyes; 45 women, 61 eyes) who underwent a surgery without use of antiglaucoma medication were identified. The types of glaucoma, glaucoma procedures, and reasons for choosing the glaucoma surgeries rather than medical therapy were collected for the 90 eyes.

**Results:**

Among the 90 eyes, primary angle-closure disease (PACD) (60%) was the most frequent type of glaucoma followed by EXG (17%), POAG (16%), and others (8%). Among the reasons for the choice of incisional surgery, relief of angle closure (64%) was the most frequent, the second most frequent was the incidental diagnosis of glaucoma during the ocular examinations both for that eye’s cataract surgery or the contralateral glaucoma surgery (13%). Other reasons included poor medication adherence (10%), dementia (6%), multiple medication allergy (3%), and acute IOP elevation other than PACD (3%). Cataract extraction (CE) alone (33%) was the most frequent glaucoma procedures performed in these eyes, followed by CE combined with goniosynechialysis (27%), CE + iStent (16%), CE + goniotomy by Tanito microhook ab interno trabeculotomy or using the Kahook Dual Blade (11%), Ahmed Glaucoma valve implantation (11%), and trabeculectomy (2%).

**Conclusion:**

In the real-world, 6.4% of incisional glaucoma surgeries were performed in the absence of medication use; of them, 32 eyes (2.3%) were with open angle glaucoma. In open angle glaucoma, the reasons can be classified into; 1) patients’ inability to instill the medication, 2) incidental diagnosis of glaucoma during the pre-surgical examinations, and 3) the eyes with acute IOP rise.

## Introduction

The standard first-line treatment for ocular hypertension and open-angle glaucomas (OAG) such as primary OAG (POAG) and exfoliation glaucoma (EXG), is topical eye drops that decrease intraocular pressure (IOP). Incisional glaucoma surgeries generally are the options considered in patients using anti-glaucoma medications. In the real world, some patients can undergo an incisional surgery without using medication, although these data are difficult to assess in the literature. We report the rate of cases with no medication treatment at the time of surgery among those that underwent incisional glaucoma surgeries performed in our department over the past 2.5 years.

## Subjects and methods

The study adhered to the tenets of the Declaration of Helsinki, and the Ethical Guidelines for Medical and Health Research Involving Human Subjects in Japan. The institutional review board of Shimane University Hospital approved the study (IRB No. 20200401–3), which did not require that each patient provide written informed consent; instead the protocol was posted at the study institution to notify participants about the study. We searched the department database of Shimane University Hospital for eyes that underwent incisional surgeries to manage glaucoma at the hospital between April 2018 and September 2020. The medical charts of 1,417 consecutive eyes listed were reviewed, and 90 (6.4%) eyes of 67 subjects who underwent a surgery without use of antiglaucoma medication were identified. The subjects for whom medications had been prescribed but who did not/could not use the medications and opted to undergo surgery also were included in the study. From the medical charts, the types of glaucoma, glaucoma procedures, and reasons for choosing the glaucoma surgeries rather than medical therapy were collected for the 90 eyes.

## Results

Subjects (22 men, 29 eyes; 45 women, 61 eyes) were a mean ± standard deviation age of 72 ± 16 years (range, 14–94 years). Among the 90 eyes, primary angle-closure disease (PACD) (60%) was the most frequent type of glaucoma followed by EXG (17%), POAG (16%), and others (8%) (Table 1). Among the reasons for the choice of incisional surgery, relief of angle closure (64%) was the most frequent, the second most frequent was the incidental diagnosis of glaucoma during the ocular examinations both for that eye’s cataract surgery or the contralateral glaucoma surgery (13%). Other reasons included poor medication adherence (10%), dementia (6%), multiple medication allergy (3%), and acute IOP elevation other than PACD (3%) (Table 1). Cataract extraction (CE) alone (33%) was the most frequent glaucoma procedures performed in these eyes, followed by CE combined with goniosynechialysis (GSL) (27%), CE + iStent (Glaukos Corporation, San Clemente, CA) (16%), CE + goniotomy by Taito microhook ab interno trabeculotomy or using the Kahook Dual Blade (New World Medical, Inc., Rancho Cucamonga, CA) (11%), Ahmed Glaucoma valve implantation (New World Medical, Inc.) (11%), and trabeculectomy (2%) (Table 1). For 12 eyes received glaucoma surgery due to the incidental diagnosis of glaucoma during the ocular examinations both for that eye’s cataract surgery or the contralateral glaucoma surgery, CE + iStent (54%) was the most frequent glaucoma procedure, followed by CE + goniotomy (38%) (Table 1). For 17 eyes received glaucoma surgery due to poor medication adherence, dementia, or multiple medication allergy, CE + iStent (35%) and Ahmed Glaucoma Valve (35%) were the most frequent glaucoma procedures, followed by CE + goniotomy (23%), and trabeculectomy (6%) (Table 1). Three eyes that received glaucoma surgery due to acute IOP rises other than PACD were treated with trabeculectomy for Posner-Schlossman syndrome, Ahmed Glaucoma Valve for sarcoidosis, and CE (+ anterior vitrectomy) for lens induced uveitis (Table 1).

**Table 1:** Summary of 90 eyes treated with glaucoma surgery without use of antiglaucoma medication

**Glaucoma type**	**n (%)**
PACD	54 (60)
Exfoliation glaucoma	15 (17)
POAG	14 (16)
Other	7 (8)
**Reason for glaucoma surgery**	
Relief of angle closure	58 (64)
Incidental glaucoma diagnosis during presurgical evaluation for cataract surgery or fellow eye's glaucoma surgery	12 (13)
Poor medication adherence other than dementia	9 (10)
Dementia	5 (6)
Multiple medication allergy	3 (3)
Acute IOP rises other than PACD	3 (3)
	(PSS 1, lens-induced uveitis 1, sarcoidosis 1)
**Surgical procedure**	
CE	30 (33)
CE + GSL	24 (27)
CE + iStent	14 (16)
CE + goniotomy	10 (11) (TMH 9, KDB 1)
Ahmed Glaucoma Valve	10 (11)
Trabeculectomy	2 (2)
**Surgical procedure for 12 cases with incidental glaucoma diagnosis during presurgical evaluation for cataract surgery or fellow eye's glaucoma surgery**	
CE + iStent	7 (58)
CE + goniotomy	5 (42) (TMH4, KDB 1)
**Surgical procedure for 17 cases with poor medication adherence other than dementia, dementia, and multiple medication allergy**	
CE + iStent	6 (35)
Ahmed Glaucoma Valve	6 (35)
CE + Goniotomy	4 (24)
Trabeculectomy	1 (6)
**Surgical procedure for 3 cases with acute IOP rises other than PACD**	
Ahmed Glaucoma Valve	1 (33)
Trabeculectomy	1 (33)
CE	1 (33)

*PACD* primary angle-closure disease, *POAG* primary open-angle glaucoma, *IOP* intraocular pressure, *PSS* Posner-Schlossman syndrome, *CE* cataract extraction, *GSL* goniosynechialysis, *TMH* Tanito microhook *ab interno* trabeculotomy; *KDB* Kahook Dual Blade

## Discussion

In our department, 6.4% of incisional glaucoma surgeries were performed in the absence of medication use or because of the patients’ inability to instill the medication. To our best knowledge, this information is unique in the literature. Based on the glaucoma type, the study eyes can be classified into two groups; the one group is PACD and the other group is open angle glaucoma. Based on the reasons for choice of surgery, most of the eyes in the latter group can be further classified into three groups; 1) the eyes of the patients with poor tolerance/adherence to medication therapy, 2) the eyes of the patients with incidental diagnosis of glaucoma during the pre-surgical examinations for cataract or opposite eye’s glaucoma surgery, and 3) the eyes with acute IOP rise that required immediate IOP lowering surgery such as secondary open angle glaucoma due to inflammation.

In this study, the most frequent types of glaucoma and surgical procedure were the PACD and CE alone, respectively. CE has been considered a first-line treatment in PACD [1], and the combination of GSL and CE may be more effective than CE alone for lowering IOP in PACD [2]. Accordingly, the high frequencies of relief of angle closure as the surgical indication and CE + GSL/CE alone as surgical procedures reflect the current treatment recommendations. The reasons for choice of CE alone, CE + GSL, or CE + minimally invasive glaucoma surgery (MIGS) would be interesting to know and is needed to be clarified in the future.

Previous studies have reported that poor medication adherence and drug-induced side effects, e.g., ocular surface diseases and drug allergy, were related to poor visual prognosis in glaucoma [3, 4]. Conditions associated with older age were anticipated to reduce medication adherence due to difficulty instilling eye drops (e.g., tremor), difficulty in accessing educational resources (e.g., lack of internet access), and reduced treatment motivation (e.g., dementia and depression) [5, 6]. In the current study, 17 eyes (19%) underwent glaucoma surgery due to poor adherence or intolerance to medication; these were the major reasons for glaucoma surgery in OAG. With this scenario, to provide adherence-independent reduction of IOP, filtration surgeries such as tube shunt surgery or trabeculectomy tended to be performed (Table 1).

Previous studies have reported that up to 90% of patients were unaware of their diagnosis when they were diagnosed [7, 8]. Accordingly, in subjects with cataract, glaucoma can be diagnosed initially during the surgical examinations before cataract surgery. In the same context, in subjects with unilateral glaucoma, glaucoma can be diagnosed initially in the contralateral eye during the surgical examinations before glaucoma surgery for the other eye. For this group of eyes, MIGS including use of the iStent and goniotomy were the choices of surgical procedures, (Table 1). MIGS have gained popularity because of the ease of performing them during CE, the safer profile than filtration surgeries, and effective reduction of the numbers of antiglaucoma medications needed [9–11]. Among the current cases, the incidentally identified glaucoma with visually significant cataract were all treated by MIGS + CE. Given the study site is the tertiary care hospital, and the patients refer from far and wide area. For such patients, especially for older patients, specialized glaucoma care is sometimes difficult to access in their local area. In Japan, many of the cataract surgery and most of the glaucoma surgery are done by hospitalizations. Accordingly, patients of this group likely choose primary glaucoma surgery because they saw the hospitalization as a good opportunity to receive specialized glaucoma treatment.

It is important to note that the data shown in this study and interpretation of data are strictly related to the country and region where the patients live, and the roles of hospital where the physicians work. Ethnic, social, and economic differences or factors should affect the data, too. Insufficiency/absence of the analyses between the relationships between surgical procedure/reasons for surgical choice and the background characteristics (i.e., age, sex, glaucoma stage and severity, etc.…) are the weak point of this study. However, we believe that the purpose of this study, that is, first to elucidate the rate of incisional glaucoma surgeries performed in the absence of medication use by entire number survey, is achieved.

Based on the vital statistics of the Japanese Government, the aging rate of the population 65 years old or older in Japan is the highest worldwide (http://www.cao.go.jp/en/whitepaper.html); therefore, we expect that the choice of glaucoma surgery rather than medication therapy due to age-related reduction in medication adherence is going to increase in the future in areas of the world where individuals will live to an advanced age.

## Data Availability

The datasets generated and/or analyzed during the current study are not publicly available due to limitations of ethical approval involving the patient data and anonymity but are available from the corresponding author on reasonable request.
